# School Term *vs.* School Holiday: Associations with Children’s Physical Activity, Screen-Time, Diet and Sleep

**DOI:** 10.3390/ijerph120808861

**Published:** 2015-07-30

**Authors:** Amanda E. Staiano, Stephanie T. Broyles, Peter T. Katzmarzyk

**Affiliations:** Pennington Biomedical Research Center, 6400 Perkins Road, Baton Rouge, LA 70808-4124, USA; E-Mails: stephanie.broyles@pbrc.edu (S.T.B.); peter.katzmarzyk@pbrc.edu (P.T.K.)

**Keywords:** weather, humidity, temperature, health behavior, sedentary lifestyle, child, adolescent

## Abstract

This cross-sectional study examined differences in children’s health behaviors during school term (ST) *versus* school holiday (SH: June–July) and how associations changed when weather characteristics were considered. Children aged 5–18 years (n = 406) from a subtropical climate reported behaviors over 20 months. Multivariable regression models controlling for age, sex, race and body mass index z-score（BMIz ） were used to examine associations between SH and each behavior. A second model included heat index, precipitation and daylight hours. Strenuous activity, moderate activity, total activity and TV viewing were significantly higher during SH than ST. After adjusting for weather characteristics, total activity remained significantly higher during SH, but the association with TV viewing was attenuated. Youth surveyed during high precipitation were significantly less likely to meet physical activity guidelines. There were no significant associations between SH and meeting sleep, physical activity or screen-time guidelines. Weather characteristics influenced associations between SH and youth’s physical activity and TV viewing.

## 1. Introduction

Children typically gain weight at a more rapid pace during school holiday than during the school term; this pattern is particularly evident among children who are overweight [1,2] and African American [2,3]. School holiday in the United States typically occurs between the transition from one grade level to the next in the months of June and July. It is unclear the extent to which weather conditions drive children’s health behaviors and subsequent weight gain during school holiday. Temperature and precipitation are prevailing influences on health behaviors, and both questionnaire and objective data indicate that physical activity among the general population increases from cold weather to warmer weather [4]. In colder climates, warmer weather with low precipitation is related to higher levels of moderate to vigorous physical activity among adolescents [5]. In contrast, June and July in North American subtropical climates are characterized by high humidity, temperature and precipitation, potentially discouraging outdoor physical activity. Furthermore, children driven inside during the out-of-school time may consume more foods and engage in more screen-time behavior, contributing to potential weight gain.

The purpose of this study was to examine differences in physical activity, dietary behaviors, screen-time and sleep between the school term and the school holiday among children living in a subtropical climate. It was hypothesized that children’s health behaviors during school holiday are less favorable than during the school term due to the adverse weather conditions.

## 2. Experimental Section

### 2.1. Research Design

A cross-sectional examination of children and adolescents was conducted. Overall, 423 5–18-year-old children and adolescents participated in one clinic visit between January 2010 and August 2011. Participants were recruited over a 20-month period (January 2010–August 2011) from the greater Baton Rouge area in south Louisiana, a humid subtropical climate with hot, humid summers and short, mild winters, located in the southeastern region of the United States. Participants were telephone screened in an attempt to achieve a balanced sample of boys and girls, of whites and African Americans and of normal weight, overweight and obese status.

### 2.2. Sample

Participants were excluded from the present analyses if they were missing dietary information (n = 4), or sleep information (n = 6), or if the child’s home residence was not in the parish (county) or a contiguous parish where the research laboratory was located and where the weather data were collected. The resulting analytical sample consisted of 406 participants aged 12.1 ± 3.6 years old (52% female, 51% African American, 46% white, 3% other). All study procedures were approved by the Pennington Biomedical Research Center’s Institutional Review Board. Participants provided written assent (consent when 18 years old), and parents provided written informed consent for minors.

### 2.3. Measures

Weather: Daily humidity, temperature, wind speed and precipitation were collected from Weather Underground (http://www.wunderground.com), based on data from the U.S. National Weather Service’s National Digital Forecast Database. Average heat index, wind chill and precipitation were calculated based on the prior 7 days and 30 days of each participant’s clinic visit. Monthly average daylight hours were recorded from the U.S. Naval Observatory’s Astronomical Applications Department (http://aa.usno.navy.mil).

Anthropometry: Using a GSE 460 digital scale (Livonia, Michigan, U.S.), weight was measured to the nearest 0.1 kg with outer clothing and shoes removed. Using a wall-mounted Harpenden stadiometer (Holtain Limited, Crosswell, Crymych, United Kingdom), height was measured to the nearest 0.1 cm. Weight and height were each averaged from two measurements or the closest two of three if measurement difference exceeded 0.5 kg for weight or 0.5 cm for height. Body mass index z-score (BMIz) was calculated using the United States Centers for Disease Control and Prevention (CDC) growth chart SAS macro program [6].

Lifestyle behaviors: Participants completed a lifestyle questionnaire, using parental assistance when necessary. Physical activity was self-reported using the Godin-Shephard Leisure-Time Physical Activity Questionnaire [7], which is a reliable instrument that has been validated against the Caltrac accelerometer [8]. Participants reported bouts/week of >15 min of strenuous, moderate or mild physical activity during free time and days/week of ≥1 h of moderate to vigorous physical activity (MVPA) in a typical week (*i.e.*, “Considering a 7-day period (a week), how many times on average do you do the following kinds of exercise for more than 15 minutes during your free time (including recess)?”). Activity units per week were assigned as 9 units for each strenuous activity bout, 5 units for each moderate activity bout and 3 units for each mild activity bout [7].

Youth self-reported consumption frequency of breakfast (number of weekdays and weekend days) using validated questions from the Health Behaviour in School-Aged Children Survey [9], as well as meals prepared away from home (total number/week) and fast food meals (total number/week), in a week. Youth self-reported hours/day of sleep, TV viewing and computer use in the prior 30 days, using adapted questions from the U.S. National Health and Examination Survey. Participants were classified as meeting or not meeting national guidelines for physical activity (≥1 hour/day MVPA) [10], screen-time (≤2 hours/day TV or computer use) [11] and sleep (≥10 hours/day for 5–12-year-olds, ≥9 hours/day for 13–18-year-olds) [12].

### 2.4. Statistical Analysis

Because wind chill is only calculated when temperatures are ≤10 °C and these temperatures did not occur during school holiday, wind chill was not used in the analyses. *T*-tests and chi-square analyses were used to compare youth surveyed during school holiday (June and July, n = 121) *vs.* youth surveyed during the school term (August–May, n = 285). Multivariable linear regression analysis controlling for age, sex, race and BMIz was used to examine the association of school holiday (1 = school holiday, 0 = school term) with self-reported lifestyle habits. In a second model, heat index, precipitation and daylight hours were used to examine the associations with self-reported lifestyle habits. The average daily heat index and precipitation in the prior 7 days of each participant’s clinic visit was used for the 7-day recall variables (physical activity and diet), and averages in the prior 30 days of each clinic visit were used for the 30-day recall variables (screen-time and sleep). The proportion of variance explained (adjusted R^2^) is reported. Logistic regression controlling for age, sex, race, BMIz and daylight hours was used to examine the association between being surveyed during high heat index and high temperature (top quartile of each weather index of the weeks surveyed for physical activity and months surveyed for screen-time and sleep) with the likelihood of meeting physical activity, screen-time and sleep guidelines. Sensitivity analyses were performed to exclude participants who were measured close to holiday breaks during the last week of November through the last week of January (n = 30).

## 3. Results

Youth surveyed during school holiday *vs*. school term did not vary by sex, race or BMIz, but were significantly older (12.8 *vs.* 11.8 years of age). Descriptive characteristics of the youth surveyed and weather characteristics stratified by school holiday *vs*. school term are reported in Table 1. Heat index, precipitation, humidity and temperature were significantly higher during the school holiday, and the school holiday had more hours of daylight than the school term. Weeks during the school holiday were also more often in the top quartile for the heat index and precipitation.

**Table 1:** School holiday *vs.* school term: characteristics of youth and weather.

	School Holiday (n = 121)		School Term (n = 285)	
	Mean ± SD	Median (IQR)	Mean ± SD	Median (IQR)
**Youth Characteristics**				
Age	12.8 ± 3.5 ^**^	-	11.8 ± 3.6	-
Female, %	47.9	-	54.4	-
Race/Ethnicity				
African American, %	56.2	-	48.8	-
White, %	40.5	-	48.4	-
Other, %	3.3	-	2.8	-
BMI, z-score	1.0 ± 1.2	-	1.0 ± 1.1	-
Underweight, %	1.7	-	1.8	-
Normal Weight, %	47.9	-	48.4	-
Overweight, %	15.7	-	14.4	-
Obese, %	34.7	-	35.4	-
Physical Activity, days/week	3.4 ± 2.2	-	3.2 ± 2.1	-
Strenuous Activity, bouts/week	4.5 ± 7.0 ^*^	3 (0, 5)	3.2 ± 3.3	3 (0, 5)
Moderate Activity, bouts/week	4.7 ± 5.1 ^*^	3 (0, 7)	3.7 ± 3.1	3 (1, 5)
Mild Activity, bouts/week	4.1 ± 5.2	2 (1, 7)	3.8 ± 3.6	3 (1, 7)
Activity units/week	76.2 ± 95.0 ^*^	49 (26, 85)	59.1 ± 41.9	54 (31, 80)
Met Guidelines, %	14.0	-	8.0	-
Screen-Time				
TV Viewing, hours/day	3.6 ± 1.5 ^**^	4 (2, 5)	3.1 ± 1.6	3 (2, 5)
Computer Use, hours/day	2.6 ± 1.9	2 (1, 5)	2.4 ± 1.9	2 (1, 4)
Met Guidelines, %	12.4	-	18.3	-
Dietary Pattern				
Breakfast, days/week	5.7 ± 1.9	7 (5, 7)	5.9 ± 1.7	7 (5, 7)
Breakfast Weekdays, days/week	4.1 ± 1.5	5 (4, 5)	4.2 ± 1.4	5 (4, 5)
Prepared Meals, meals/week	3.3 ± 3.5	2 (1, 4)	2.8 ± 2.4	2 (1, 4)
Fast Food Meals, meals/week	2.0 ± 2.0	2 (1, 3)	1.8 ± 2.0	1 (1, 3)
Sleep, hours/day	8.5 ± 1.3	9 (8, 9)	8.5 ± 1.6	8 (8, 9)
Met Guidelines, %	39.7	-	28.4	-
**Weather Characteristics ^a^**				
Heat Index	82.5 ± 5.4 ^***^	-	57.5 ± 14.2	-
Top Quartile Heat Index, %	62.0 ^***^	-	9.1	-
Precipitation, cm	0.6 ± 0.4 ^***^	-	0.4 ± 0.6	-
Top Quartile Rainfall, %	36.4 ^**^	-	21.8	-
Daylight, hours	14.0 ± 0.1 ^*^	-	12.3 ± 1.1	-
Humidity, %	74.6 ± 4.6 ^***^	-	71.7 ± 6.4	-
Temperature, °C	28.7 ± 0.9 ^***^	-	21.8 ± 6.6	-

Notes: ^*^ *p* < 0.05, ^**^ *p* < 0.01, ^***^ *p* < 0.001 difference between school term and school holiday; **^a^** weather characteristics are reported as daily average during prior 7 days.

In multivariable linear regression analyses adjusted for age, sex, race and BMIz, school holiday was positively associated with more bouts/week of strenuous (*p* = 0.02) and moderate activity (*p* = 0.005), higher activity units/week (*p* = 0.009) and more hours/day of TV viewing (*p* = 0.008) (see Table 2 and Table 3). Compared to those surveyed during the school term, children surveyed during school holiday on average obtained one more bout of >15-min strenuous activity per week and one more bout of >15-min of moderate activity per week, and this contributed to 17.8 more activity units per week. Children surveyed during the school holiday on average viewed television for 0.5 hours more per day.

In Model 2, which also included heat index, precipitation and daylight hours, children surveyed during school holiday still obtained significantly more activity units than those surveyed during school term (*p* = 0.045). However, the other significant associations with school holiday were attenuated to non-significance. Heat index was negatively associated with weekday breakfast consumption (*p* = 0.038). Daylight hours were positively associated with days/week of breakfast (*p* = 0.008). The fully-adjusted models explained 17% of the variance in breakfast consumption, 16% of the variance in sleep, 9% of the variance in television viewing and ≤5% of the variance in the other health behaviors.

In logistic regression analyses that included weather indicators (see Table 4), being surveyed during the school holiday was not significantly associated with the likelihood of meeting physical activity, screen-time or sleep guidelines. Youth surveyed during high precipitation were significantly less likely to meet physical activity guidelines (odds ratio = 0.3, 95% confidence interval: 0.1–0.9).

### Sensitivity Analysis

Excluding participants who attended clinic visits during the holiday season (last week of November through last week of January) did not appreciably change the results.

**Table 2:** Results of multivariable regression examining associations of school holiday with lifestyle habits over a week.

	Strenuous Activity, bouts/week		Moderate Activity, bouts/week		Mild Activity, bouts/week	
	Model 1	Model 2	Model 1	Model 2	Model 1	Model 2
**School Holiday**	1.2 ± 0.5 ^*^	1.5 ± 0.7	1.2 ± 0.4 ^**^	1.2 ± 0.6	0.5 ± 0.5	0.3 ± 0.7
**Heat Index**	-	−0.01 ± 0.02	-	−0.01 ± 0.02	-	−0.002 ± 0.02
**Precipitation, cm**	-	0.2 ± 0.4	-	−0.2 ± 0.3	-	−0.4 ± 0.4
**Daylight, hours**	-	−0.08 ± 0.3	-	0.1 ± 0.2	-	0.1 ± 0.2
**Adjusted R^2^**	0.03	0.03	0.04	0.04	0.01	0.01
	**Activity,** **units/week**		**Physical Activity,** **days/week**		**Breakfast,** **days/week**	
	**Model 1**	**Model 2**	**Model 1**	**Model 2**	**Model 1**	**Model 2**
**School Holiday**	17.8 ± 6.8 ^**^	20.0 ± 9.9 ^*^	0.2 ± 0.2	0.5 ± 0.3	0.01 ± 0.2	−0.02 ± 0.3
**Heat Index**	-	−0.1 ± 0.3	-	−0.01 ± 0.01	-	−0.01 ± 0.01
**Precipitation, cm**	-	−0.5 ± 5.8	-	−0.2 ± 0.2	-	−0.08 ± 0.1
**Daylight, hours**	-	0.2 ± 3.5	-	−0.01 ± 0.1	-	0.2 ± 0.1 ^**^
**Adjusted R^2^**	0.04	0.03	0.03	0.03	0.15	0.17
	**Breakfast Weekdays,** **days/week**		**Fast Food Meals,** **meals/week**		**Prepared Meals,** **meals/week**	
	**Model 1**	**Model 2**	**Model 1**	**Model 2**	**Model 1**	**Model 2**
**School Holiday**	0.03 ± 0.1	0.003 ± 0.2	0.1 ± 0.2	0.3 ± 0.3	0.5 ± 0.3	0.7 ± 0.4
**Heat Index**	-	−0.01 ± 0.01 ^*^	-	−0.003 ± 0.01	-	−0.002 ± 0.01
**Precipitation, cm**	-	−0.08 ± 0.1	-	−0.1 ± 0.2	-	−0.4 ± 0.3
**Daylight, hours**	-	0.2 ± 0.1 ^**^	-	−0.1 ± 0.1	-	−0.1 ± 0.2
**Adjusted R^2^**	0.13	0.15	0.03	0.02	0.02	0.02

Notes: ^*^ *p* < 0.05, ^**^ *p* < 0.01; values are estimates ± standard error; analyses controlled for age, sex, race and BMIz.

**Table 3:** Results of multivariable regression examining associations of school holiday with lifestyle habits over the prior 30 days.

	TV Viewing, hours/day		Computer Use, hours/day		Sleep, hours/day	
	Model 1	Model 2	Model 1	Model 2	Model 1	Model 2
**School Holiday**	0.5 ± 0.2 ^**^	−0.1 ± 0.2	0.05 ± 0.2	−0.3 ± 0.3	0.3 ± 0.1	0.2 ± 0.2
**Heat Index**	-	0.01 ± 0.01	-	0.01 ± 0.01	-	0.002 ± 0.01
**Precipitation, cm**	-	−0.4 ± 0.3	-	0.7 ± 0.4 ^*^	-	−0.2 ± 0.3
**Daylight, hours**	-	0.2 ± 0.1 ^*^	-	0.1 ± 0.1	-	0.03 ± 0.1
**Adjusted R^2^**	0.06	0.09	0.03	0.05	0.17	0.16

Notes: ^*^ *p* < 0.05, ^**^ *p* < 0.01; values are estimates ± standard error; analyses controlled for age, sex, race and BMIz.

**Table 4:** Results of logistic regression analyses for likelihood of meeting physical activity, screen-time and sleep guidelines, with being surveyed during school holiday, high heat index and high precipitation. MVPA, moderate to vigorous physical activity.

	Met Physical Activity Guidelines (≥1 hour/day MVPA)	Met Screen-Time Guidelines (≤2 hours/day TV or computer use)	Met Sleep Guidelines (≥10 hours/day for 5–12 year-olds, ≥9 hours/day for 13–18 year-olds)
**School Holiday**	2.6 (0.9–7.5)	1.8 (0.7–4.3)	1.9 (1.0–3.7)
**Top Quartile of Heat Index**	0.9 (0.4–2.3)	0.5 (0.2–1.2)	1.4 (0.7–2.5)
**Top Quartile of Precipitation**	**0.3** (0.1–0.9) ^*^	0.8 (0.4–1.6)	0.8 (0.5–1.4)

Note: ^*^ *p* < 0.05; ^**^ *p* < 0.01; values are the odds ratio (95th percent confidence interval); analyses controlled for age, sex, race, BMIz and daylight hours.

## 4. Discussion

Contrary to the hypothesis, children’s health behaviors were more favorable during school holiday, including more physical activity, and there were no observed dietary differences between school holiday and school term. These findings conflict with a narrative review that indicated physical activity declines across school holiday [1] and with a second study of overweight and obese youth in which energy expenditure measured by doubly-labeled water did not vary between school holiday and school term [13]. In the present study, the one behavior that was less favorable during school holiday was TV viewing, with children watching on average 30 minutes/day more TV during the school holiday (3.6 hours/day) than during the school term (3.1 hours/day). Greater discretionary time during school holiday may increase time spent in all leisure activities.

Importantly, total activity remained significantly higher in the school holiday even when weather characteristics were included in the model. However, significant differences in children’s TV viewing and in specific types of physical activity (*i.e.* strenuous and moderate) were attenuated when weather characteristics were included in the model. Indeed, children surveyed during months in the top quartile of precipitation were significantly less likely to meet physical activity guidelines. Children surveyed during school holiday faced weather challenges: months during school holiday were hotter, more humid and had more precipitation. The city of Baton Rouge where the participants lived was recently rated as the highest “urban heat island” city in the U.S. based on temperature variation between rural and urban areas, a characteristic that researchers suggest poses detriments to human health [14]. Despite these weather challenges, children’s physical activity was higher during the school holiday, suggesting that children were not deterred from outdoor play or found indoor activities.

Several mechanisms may contribute to the influence of weather on children’s behavior. For instance, physiological mechanisms affecting homeostatic regulation may affect food intake, energy expenditure and, therefore, body weight differently across seasons [15], though dietary pattern was not associated with weather characteristics in the present study. These changes may be less prominent in modern society due to indoor air-conditioning and heating options. Daily weather may impact a child’s micro-environment by influencing their choices to engage in physical activity, watch TV or use the computer and consume snacks. Daily weather may also influence the rules created by the child’s parents or caregivers, thereby facilitating or diminishing access to physical activity, screen-time and dietary options. These influences may be particularly influential for children who are overweight and susceptible to weight gain during the school holiday [1].

Childcare, family and school environments may vary when a child is attending school *versus* out of school, and these environments may differentially impact a child’s health behaviors. For instance, during school holiday children who were not involved in a structured environment (*i.e.*, summer camp) had lower physical activity and were less likely to consume breakfast than those who were involved in a structured program [16]. Proposed solutions to lessen school holiday weight gain include structured physical activity programming and access to facilities, as well as food programs [3].

This study had several strengths and limitations. A key strength is the diversity of the sample, with both African American and white boys and girls aged 5–18 years. Participants were surveyed during a 20-month period, allowing an examination of both school term and school holiday behaviors and varying weather patterns across the year. A key limitation is that these were cross-sectional data. Following children longitudinally throughout the year would allow for within-child comparisons of behavioral variances due to school holiday and weather. Because precipitation was calculated as an average across the week or month prior to each clinic visit, it is not clear from the present data how the duration of rainfall or timing during the day may affect children’s behaviors during waking hours. A second limitation is that the children provided self-reported data. Objectively-assessed physical activity using accelerometry, along with 24-hour food recall instruments to assess dietary intake would provide more accurate data. There is also the potential for misclassification. The participants were not specifically asked if they were in school or out of school, so a participant who was home-schooled or followed a non-traditional school schedule may have been classified incorrectly. However, sensitivity analysis that removed participants who were surveyed during the November–January period did not indicate differences in observed associations. Self-report questions queried about activities over the past seven days or the past 30 days, so children who were on the cusp of school holiday may have provided responses consistent with their school term activities.

Given the observed associations between weather and health behaviors, public health recommendations should include adaptations for the promotion of physical activity in adverse weather conditions, including the use of adapted clothing and equipment and providing indoor options for activities [1,4]. Given that children’s TV use was higher when the heat index was high, incorporating physical activity into screen-based entertainment may help defend against lost physical activity time. In regards to epidemiological research to examine children’s health behaviors, researchers should consider the season during which children are surveyed to account for potential weather influences on responses. Weather influences may even affect intervention outcomes. Finally, future research should investigate ways to promote healthy behaviors while protecting the child from high humidity, temperature and precipitation, in order to slow pediatric weight gain.

## 5. Conclusions

In summary, children’s physical activity and TV viewing were higher during school holiday *versus* school time, and physical activity remained higher during school holiday when weather characteristics were taken into account. High precipitation was associated with a lower likelihood of the child meeting physical activity guidelines. Weather characteristics appeared to drive differences in children’s physical activity and TV viewing between school holiday and school term.

## References

[1] Baranowski T., O’Connor T., Johnston C., Hughes S., Moreno J., Chen T., Meltzer L., Baranowski J. (2014). School year *versus* summer differences in child weight gain: A narrative review. Child. Obes..

[2] von Hippel P.T., Powell B., Downey D.B., Rowland N.J. (2007). The effect of school on overweight in childhood: Gain in body mass index during the school year and during summer vacation. Am. J. Public Health.

[3] Franckle R., Adler R., Davison K. (2014). Accelerated weight gain among children during summer *versus* school year and related racial/ethnic disparities: A systematic review. Prev. Chronic Dis..

[4] Shephard R.J., Aoyagi Y. (2009). Seasonal variations in physical activity and implications for human health. Eur. J. Appl. Physiol..

[5] Aibar A., Bois J.E., Generelo E., Bengoechea E.G., Paillard T., Zaragoza J. (2015). Effect of weather, school transport, and perceived neighborhood characteristics on moderate to vigorous physical activity levels of adolescents from two European cities. Environ. Behav..

[6] Centers for Disease Control and Prevention A SAS program for the CDC growth charts. http://www.cdc.gov/nccdphp/dnpao/growthcharts/resources/sas.htm.

[7] Godin G., Shephard R.J. (1985). A simple method to assess exercise behavior in the community. Can. J. Appl. Sport Sci..

[8] Miller D.J., Freedson P.S., Kline G.M. (1994). Comparison of activity levels using the Caltrac accelerometer and five questionnaires. Med. Sci. Sport Exerc..

[9] Roberts C., Freeman J., Samdal O., Schnohr C.W., de Looze M.E., Nic Gabhainn S., Iannotti R., Rasmussen M., for the International HBSC Study Group (2009). The Health Behaviour in School-aged Children (HBSC) study: Methodological developments and current tensions. Int. J. Public Health.

[10] Physical Activity Guidelines Advisory Committee (2008). Physical Activity Guidelines Advisory Committee Report.

[11] The American Academy of Pediatrics Council on Communications Media (2011). Policy Statement—Children, Adolescents, Obesity, and the Media. Pediatrics.

[12] National Heart, Lung, and Blood Institute How much sleep is enough?. http://www.nhlbi.nih.gov/health/health-topics/topics/sdd/howmuch.html.

[13] Zinkel S.R., Moe M., Stern E.A., Hubbard V.S., Yanovski S.Z., Yanovski J.A., Schoeller D.A. (2013). Comparison of total energy expenditure between school and summer months. Pediatr. Obes..

[14] Zhao L., Lee Z., Smith R.B., Oleson K. (2014). Strong contributions of local background climate to urban heat islands. Nature.

[15] Ebling F.J., Barrett P. (2008). The regulation of seasonal changes in food intake and body weight. J. Neuroendocrinol..

[16] Tovar A., Lividini K, Economos C.D., Folta S., Goldberg J., Must A. (2010). School’s out: What are urban children doing? The Summer Activity Study of Somerville Youth (SASSY). BMC Pediatr..

